# High-Multiplex Proteomics: Analytical Trade-Offs and a Practical Selection Framework for Emerging Platforms

**DOI:** 10.3390/proteomes14040051

**Published:** 2026-09-29

**Authors:** Owahabanun-Joshua Okojie, Ana Checa-Ros, Luis D’Marco

**Affiliations:** Grupo de Investigación en Enfermedades Cardiorrenales y Metabólicas, Departamento de Medicina y Cirugía, Facultad de Ciencias de la Salud, Universidad Cardenal Herrera-CEU, CEU Universities, 46115 Valencia, Spain

**Keywords:** proteoforms, biofluid profiling, epitope accessibility, protein inference, high-throughput screening, translational biomarkers

## Abstract

Classical liquid chromatography–tandem mass spectrometry (LC-MS/MS) remains a cornerstone of proteomics, but deep profiling of complex biofluids is constrained by the extreme dynamic range of protein abundance and low sample throughput. Emerging high-multiplex platforms address these limitations through distinct high-throughput configurations, including antibody-based proximity assays, modified aptamers, nanoparticle-assisted mass spectrometry, and digital single-molecule immunoassays. Despite their discovery potential, these technologies operate within alternative bottom-up or affinity-based frameworks, meaning that they are not interchangeable. Quantitative measurements of nominally identical proteins frequently show poor to moderate cross-platform agreement, reflecting fundamental differences in chemistry, epitope accessibility, matrix susceptibility, genetic variants, and peptide-level protein inference. Crucially, because these platforms generally yield aggregate quantitative readouts, they typically average across the proteoform spectrum, limiting their ability to differentiate whether an abundance shift is driven by specific splice variants, localized post-translational modifications, or a uniform increase in the protein’s overall concentration. This review provides a balanced, decision-oriented comparison of the Olink Proximity Extension Assay, SomaLogic SomaScan, Seer Proteograph and Quanterix Simoa, delineating their operational utility alongside their respective technical limitations. We propose a practical framework to guide platform selection based on the specific biological question, cohort size, and required structural resolution, while evaluating strategies to enhance cross-platform reproducibility via standardized reference matrices, structure-informed interpretation, and multi-tiered validation workflows.

## 1. Introduction

Classical liquid chromatography–tandem mass spectrometry (LC-MS/MS) has long served as a foundational discovery technology in proteomics [1]. Modern MS workflows can provide broad peptide- and protein-level profiles and, in appropriately designed assays, quantitative information across complex biological matrices [1,2]. However, translational studies of plasma, serum, urine, and cerebrospinal fluid (CSF) face severe analytical bottlenecks [3]. Informative biomarkers frequently span many orders of magnitude in concentration, and profiling large clinical cohorts requires a level of sample throughput that exceeds the operational capacity of conventional MS workflows [3].

A central challenge in biofluid proteomics is the extreme dynamic range of protein abundance [4]. In plasma, a small number of highly abundant proteins, primarily albumin and immunoglobulins, account for most of the total protein mass, whereas cytokines, signaling molecules, and tissue-leakage markers circulate at much lower concentrations [5].

Traditional discovery MS addresses this imbalance through multidimensional fractionation, immunoaffinity depletion, or peptide-level enrichment [3,6]. While these approaches successfully expand proteome depth, they inherently introduce non-specific sample loss, increase analytical runtimes, and add technical variability [7,8]. Consequently, deploying deep conventional bottom-up MS to very large clinical cohorts remains constrained by cost and instrument capacity [9].

These throughput and scaling limitations have driven the development and commercial adoption of high-multiplex proteomics platforms. Diverse technological configurations, including antibody-based proximity assays, modified aptamers, nanoparticle-assisted mass spectrometry, and digital single-molecule immunoassays utilize distinct biochemical architectures to increase analytical throughput and, depending on the platform, improve access to lower-abundance protein targets [10,11,12,13]. Affinity-based systems convert protein-recognition events into amplifiable nucleic-acid reporters, digital single-molecule immunoassays isolate protein-bead complexes, and nanoparticle arrays utilize specialized surface coatings to bind and concentrate low-abundance protein subsets prior to automated downstream LC-MS/MS [14,15,16,17]. Consequently, these automated configurations permit the high-throughput screening of thousands of protein targets across large scale cohort studies, enabling large-scale biomarker association analyses [8].

Operating on alternative bottom-up or affinity-based frameworks prevents direct profiling of whole proteins [18]. As a result, the analytical readout is limited to a pre-specified subset, set by proprietary affinity menus or local surface-binding kinetics. This high-throughput scaling ultimately compromises proteoform resolution [18]. The human proteome encompasses a complex spectrum of proteoforms, including splice variants, cleavage products, and varied post-translational modifications, which constitute the molecular species responsible for specific cellular mechanisms [19]. Current high-multiplex screening platforms typically generate an aggregate quantitative signal, thereby blending variations across the proteoform spectrum of a target protein [18,20]. Consequently, they lack the resolution to distinguish between an association driven by a single specific proteoform and one resulting from a uniform shift in the protein’s overall concentration baseline [20]. 

These structural and analytical differences also contribute to challenges in cross-platform harmonization and standardization [21,22]. Quantitative values generated for nominally identical proteins exhibit poor to moderate correlation across platforms, a discrepancy heavily influenced by variations in recognition chemistry, epitope accessibility, matrix interference and hidden structural genetic variants [22,23]. Treating high-throughput screening data as definitive without evaluating these structural constraints can lead to false-positive target prioritization and compromised biomarker validation [20,22]. This review provides a critical, decision-oriented evaluation of these emerging formats, using the Olink Proximity Extension Assay, SomaLogic SomaScan, Seer Proteograph, and Quanterix Simoa platforms as central case studies. Rather than treating these high-multiplex platforms as interchangeable profiling systems, we evaluate the distinct biochemical mechanisms and inherent biases characterizing each technology, framing them within the broader challenges of bottom-up protein inference and proteoform diversity. Additionally, a practical selection framework is proposed to align platform features with specific research objectives, followed by a discussion of methodological priorities for platform harmonization, validation and proteoform mapping.

## 2. Overview of High-Multiplex Proteomics Platforms

### 2.1. Olink: Proximity Extension Assay (PEA)

The Proximity Extension Assay (PEA) framework mitigates antibody cross-reactivity in high-multiplex immunoassays by relying on dual-epitope recognition [16]. The assay employs pairs of target-specific antibodies conjugated to complementary oligonucleotide tags, where productive signal generation depends on the simultaneous binding of both probes to the target protein (Figure 1).

When both antibodies occupy their respective epitopes on a single target protein, the attached oligonucleotides are brought into spatial proximity. Their DNA sequences then hybridize and undergo enzymatic extension to generate a target-specific double-stranded DNA reporter that is subsequently amplified and quantified [16].

This dual-recognition mechanism serves as an operational specificity filter; isolated binding by a single probe fails to initiate the complete hybridization-extension cascade [24]. While this design minimizes non-specific and cross-reactive background signals, it does not completely eliminate them. Depending on the assay configuration, these reporter molecules are quantified by quantitative polymerase chain reaction (qPCR) or next-generation sequencing (NGS) [16]. The high performance and quantitative robustness of this setup using standard matrix reference materials was recently confirmed independently [25].

The primary operational characteristics of the PEA framework include low sample-volume requirements, high throughput scaling, and dual-antibody recognition [16,24]. However, as with alternative affinity-based platforms, target measurement remains fundamentally dependent on reagent performance, epitope accessibility, matrix susceptibility, and a predefined analyte menu [20,22]. As illustrated in Figure 1, these structural constraints must be accounted for when interpreting platform-specific associations, as localized alterations in epitope exposure can distort quantitative readouts independently of total protein abundance [20].

### 2.2. SomaLogic: SomaScan Assay

The SomaScan platform utilizes highly parallelized, aptamer-based recognition arrays for large-scale biofluid profiling [15]. Rather than employing traditional antibodies, the architecture relies on chemically modified single-stranded DNA aptamers designated as SOMAmers (Slow Off-rate Modified Aptamers) [15]. These synthetic oligonucleotides are engineered with base-position functional groups that mimic hydrophobic amino-acid side chains, substantially increasing their structural binding affinities and specificity for native, non-denatured protein targets (Figure 2A) [26].

The assay protocol operates through a controlled, multi-step equilibrium binding and purification sequence, utilizing synthetic SOMAmers labeled with a biotin group, a photo-cleavable linker, and a fluorescent tag [15,26]. This partitioned workflow encompasses four sequential phases.

1. Catch-1: Biotinylated SOMAmers are immobilized on streptavidin-coated solid-phase matrices and incubated with the biofluid sample to initiate protein-SOMAmer complex assembly [15].
2. Photo-Cleavage: Non-specifically bound and unbound matrix proteins are removed via stringency washes, followed by the application of ultraviolet (UV) light to cleave the photo-reactive linker, releasing the intact complexes back into solution [15].
3. Catch-2: The exposed surface residues of the released target proteins undergo manual or automated biotinylation, allowing the intact complexes to be recaptured onto a secondary streptavidin-functionalized substrate [15].
4. Elution & Quantitation: Denaturing conditions are introduced to disrupt the specific non-covalent SOMAmer-protein interactions. The liberated single-stranded SOMAmer DNA molecules, which serve as direct, stoichiometric surrogates for the original target proteins, are subsequently isolated and quantified via hybridization microarrays or NGS [15].

The primary operational advantages of the SomaScan architecture are its expansive multiplexing capacity and scalability [15]. The current 11K configuration permits the parallel measurement of approximately 11,000 protein targets from a 55 µL biofluid sample volume, facilitating population-scale discovery workflows [12,15,27]. This screening breadth, however, remains bound to a predetermined analyte menu [20,22]. Furthermore, as illustrated in Figure 2, quantitative metrics are highly sensitive to sequence conservation within the target binding region [28]. Protein-altering variants (such as single-nucleotide polymorphisms or *cis*-pQTLs) can structurally alter target epitopes, disrupting affinity kinetics and leading to false-negative quantitative readouts independently of actual circulating protein abundance [20,28].

### 2.3. Nanoparticle Protein Corona Liquid Chromatography–Mass Spectrometry

An alternative analytical paradigm bypasses predefined target-specific affinity reagents entirely, replacing them with nanoparticle-assisted physicochemical enrichment coupled to downstream mass spectrometry detection [14,29]. This approach utilizes engineered nanoparticles functionalized with distinct surface coatings to adsorb complex biofluid matrices, forming a localized biomolecular corona (Figure 3A). While multiple emerging platforms leverage this surface-adsorption phenomenon to compress total protein dynamic range before downstream LC-MS/MS analysis, the Seer Proteograph Product Suite represents the dominant commercial platform pioneering this framework today [29].

The composition of this biomolecular corona is strictly governed by baseline protein abundance, competitive binding kinetics, and nanoparticle surface chemistry [29,30,31]. While this parallel surface-adsorption process enriches low-abundance protein subsets that are typically inaccessible in unenriched biofluid samples, the framework is biased by the specific physicochemical properties of the engineered nanoparticle panel [31].

Deploying multiple nanoparticle chemistries in parallel successfully broadens proteome sampling depth and mitigates dominance by highly abundant plasma proteins [29,31]. Following corona isolation, the bound proteins undergo on-particle enzymatic cleavage, after which the resulting tryptic peptides are analyzed via LC-MS/MS characterization (Figure 3A).

The principal operational characteristic of this framework is reagent-independent discovery coupled directly to peptide-level MS readouts [29,31]. This architecture enables detection beyond predefined antibody or aptamer menus, providing complementary information regarding isoforms or protein-altering variants [14,29]. However, as illustrated in Figure 3**,** these advantages are counterbalanced by specific system constraints, including dependence on downstream MS performance, nanoparticle-specific enrichment bias during corona formation (Figure 3A), and the structural loss of intact macromolecular connectivity inherent to bottom-up tryptic digestion (Figure 3B) [20,31].

### 2.4. Digital Single-Molecule Immunoassays

Digital single-molecule immunoassay arrays provide exceptional analytical sensitivity for targeted biomarker quantification [17,32]. The framework retains the classical biochemical logic of a dual-antibody sandwich immunoassay but compartmentalizes signal detection within miniaturized arrays of femtoliter-scale wells, allowing individual enzyme-labeled bead events to be digitally counted [13,17]. While various high-sensitivity microfluidic and bead-based protein arrays are expanding in clinical utility, the Quanterix Single-Molecule Array (Simoa) platform serves as the premier commercial representative of this single-molecule isolation approach [13].

Target analytes are isolated using magnetic microbeads functionalized with specific capture antibodies. These solid-phase matrices are subsequently exposed to a biotinylated detection antibody and an enzyme conjugate (streptavidin-beta-galactosidase) to assemble an enzyme-labeled sandwich complex (Figure 4A) [13,17]. The operational architecture relies on a specialized optical loading disk containing over 200,000 micro-wells, with dimensions precisely engineered to accommodate exactly one individual magnetic micro-bead [13,17].

Following bead loading into the array, a fluorogenic substrate is introduced, and the micro-wells are sealed with oil. Because the volume of each micro-well is restricted to approximately 40 femtoliters, even a single trapped enzyme molecule rapidly converts the substrate into a highly concentrated, localized fluorescent signal [13,17].

An optical imaging system subsequently scans the array. At low target concentrations, the system executes a digital counting mechanism: micro-wells containing an enzyme-labeled sandwich complex generate a fluorescent signal (“On”), while empty or unreacted wells remain dark (“Off”). The ratio of active wells correlates directly with target concentration [13,17,33].

The primary operational advantage of this digital architecture is its capacity to quantify selected ultra-low-abundance biomarkers at sub-picogram concentrations, with assay-dependent limits of detection extending into the femtogram-per-milliliter range [17,34]. This sensitivity makes the platform highly effective for the targeted detection of these markers within highly diluted peripheral biofluids [34]. However, its multiplexing capacity remains tightly constrained compared to broad discovery screening platforms [22]. Furthermore, as illustrated in Figure 4B, its data streams are highly vulnerable to matrix interference [18,22]. Endogenous biofluid components, such as circulating autoantibodies or heterophilic factors, can sterically block or competitively obstruct epitope accessibility, disrupting secondary detection antibody assembly and generating false-negative digital readouts independently of total protein abundance [20,22].

## 3. Comparative Analysis of High-Multiplex Proteomics Platforms

The added value of high-multiplex proteomics is therefore not simply an increase in the number of proteins measured per sample. Its principal contribution is the ability to generate standardized, scalable molecular measurements across large cohorts using limited sample volumes, thereby enabling population-scale biomarker discovery and association analyses that would be difficult to achieve with conventional deep LC-MS/MS workflows. This operational advantage, however, must be weighed against platform-specific limitations in molecular specificity, proteoform resolution, and cross-platform reproducibility.

### 3.1. Multiplexing Capacity

The SomaScan platform currently provides an expansive affinity-based menu, with approximately 11,000 protein targets in the 11K assay configuration. Its principal added value is therefore population-scale breadth and sample-efficient multiplexing, rather than proteoform-resolved measurement, while the Olink PEA parallelizes the measurement of over 5400 analytes [10,12]. Its added value lies in combining high multiplexing with very low sample consumption and a dual-recognition architecture. This spatial configuration yields a substantial surge in analytical confidence; by mandating that two discrete antibodies independently bind adjacent epitopes on the same target molecule before generating an amplifiable signal, the framework effectively nullifies single-antibody cross-reactivity and non-specific background signals. This makes it particularly useful when cohort size and limited biofluid availability are major constraints. However, this operational advantage does not eliminate dependence on epitope accessibility, reagent performance, or matrix effects. Conversely, the Seer Proteograph system introduces a reagent-independent paradigm for discovery multiplexing; proteome coverage depth is dictated by localized nanoparticle surface enrichment, instrument acquisition parameters, and bioinformatic protein inference rather than a predetermined reagent menu [11,30]. Shifting from broad screening to targeted verification, the Quanterix Simoa platform is engineered as a low-plex, ultra-sensitive workflow optimized for precise low-abundance quantification [13]. Because commercial platform specifications continuously evolve, Table 1 outlines representative current configurations and operational parameters rather than fixed architectural limits.

### 3.2. Sensitivity and Dynamic Range

The Quanterix Simoa platform typically achieves the lowest analytical detection limits among the four platforms for validated targeted assays, resolving sub-picogram and, for selected analytes, femtogram-per-milliliter thresholds [17,34]. The Olink PEA and SomaScan provide high analytical sensitivity across their broad predefined reagent menus [10,12]. Conversely, the effective lower detection limits of the Seer Proteograph framework remain coupled to localized nanoparticle surface enrichment factors, downstream LC-MS/MS instrument configuration, and target-specific peptide ionization characteristics [11]. Accordingly, direct cross-platform numerical sensitivity comparisons require cautious interpretation, as concentration definitions, calibration strategies, and analytical assay metrics vary substantially [35]. Beyond lower detection thresholds, cross-platform numerical discrepancies are deeply driven by fundamental differences in absolute quantitative accuracy and the underlying biophysical constraints of the affinity reagents. Independent spike-in evaluations using standardized reference materials (such as NIST SRM 1950) have demonstrated that the dual-antibody proximity extension architecture maintains robust technical linearity and true quantitative accuracy across broad concentration scales [25]. Finally, equivalent rigorous absolute quantitative validation is more constrained for ultra-high-multiplex aptamer microarrays. This discrepancy is partly explained by the foundational binding kinetics of single-stranded oligonucleotides; recent single-molecule fluorescence evaluations demonstrate that individual aptamers frequently exhibit a highly restricted intrinsic quantitative dynamic range, often spanning fewer than two orders of magnitude [36]. When deployed across broad clinical cohorts, these localized biophysical boundaries compress absolute abundance readouts and artificially distort cross-platform mathematical concordance, even when the underlying biological concentrations remain identical.

### 3.3. Sample Volume and Throughput

Sample volume requirements diverge significantly across these platforms and operational configurations. The Olink PEA requires approximately 2 µL of plasma or serum, whereas the SomaScan 11K configuration utilizes roughly 55 µL [10,12]. Standard Seer Proteograph workflows utilize approximately 240 µL of plasma, although validated micro-volume protocols have been introduced [11]. Conversely, Quanterix Simoa volume configurations are assay-dependent and typically span the tens-of-microliters range [13]. Crucially, these nominal consumption metrics should be evaluated alongside multi-matrix batching strategies, sample dilution matrices, replicate testing requirements, quality control designs, and the absolute biofluid volume reserved for downstream orthogonal validation workflows [35,37].

### 3.4. Binding and Sampling Bias

Every high-multiplex platform exhibits intrinsic biochemical or physicochemical biases. The Olink PEA, SomaScan, and Quanterix Simoa architectures depend fundamentally on predefined affinity reagents, leaving them susceptible to alterations in epitope accessibility, target protein conformation, protein-altering sequence variants, and matrix interference factors [38,39]. Conversely, the Seer Proteograph framework bypasses target-specific antibody or aptamer panels entirely but introduces a distinct selection mechanism driven by the physicochemical surface-adsorption properties governing biomolecular corona assembly on the nanoparticle panel [40]. Consequently, untargeted proteomics discovery should be defined as reagent-independent rather than completely free of analytical selection [41]. Because these operational architectures continuously evolve, exact performance parameters, sensitivity thresholds, and biofluid input bounds remain strictly assay- and workflow-dependent.

### 3.5. Cross-Platform Concordance and Technical Reproducibility

Beyond theoretical architectural constraints, published evidence has identified important challenges in target specificity, analytical precision and cross-platform concordance of high-multiplex proteomics platforms [18,20,22,23,42,43]. Large-scale, head-to-head empirical evaluations profiling identical clinical biobank samples across platforms reveal a profound divergence in quantitative readouts [42]. Specifically, multi-platform comparisons between the Olink and SomaScan platforms demonstrate that Spearman rank correlation coefficients (ρ) for nominally identical proteins vary widely, frequently clustering at poor-to-moderate values (median rho approx. 0.45) [42]. This divergence is highly target-specific rather than uniform across the proteome. For example, large-scale empirical benchmarking in the Atherosclerosis Risk in Communities (ARIC) cohort revealed that while certain cardiovascular biomarkers such as growth differentiation factor 15 (GDF15) and interleukin-1 receptor-like 1 (ST2) exhibit high cross-platform correlation (r ≥ 0.80), key inflammatory and matrix markers like interleukin-6 (IL-6) achieve only modest correlation (0.50 ≤ r < 0.80) [44]. More strikingly, critical clinical analytes including interleukin-10 (IL-10), tumor necrosis factor-alpha (TNF-α), metalloproteinase inhibitor 1 (TIMP-1), and vascular cell adhesion protein 1 (VCAM-1) demonstrate highly discordant or very poor correlations (r < 0.50) [44]. When cross-referenced against traditional gold-standard clinical immunoassays, Olink and SomaScan exhibit distinct baseline deviations; for instance, Olink tracking of GDF15 correlates more tightly with clinical assays (ρ = 0.907) than its SomaScan counterpart (ρ = 0.791) [42]. This target-specific data discordance underscores that platforms are not trivially interchangeable. This numerical discrepancy is heavily driven by distinct platform-specific precision characteristics. For instance, recent benchmark data evaluating high-multiplex upgrades indicates that while the SomaScan 11K assay delivers superior measurement precision (median CV approx. 6.8%), the Olink PEA platform exhibits vastly greater technical variability (median CV approx. 35.7%) before strict filtering of values below the limit of detection (LOD) [42,43].

Importantly, differences in cross-platform reproducibility may also reflect platform-specific target-recognition and specificity issues. Genetic validation frameworks leveraging genomic data (*cis*-pQTL mapping) have shown that some protein–disease associations discovered via aptamer or single-antibody screening represent false positives [45]. These phenotypic signals are often completely non-overlapping across platforms due to cross-reactive recognition chemistries, hidden structural genetic variants, or matrix-induced epitope masking [45,46]. Consequently, treating high-multiplex screening datasets as definitive molecular proxies without referencing peer-reviewed multi-platform validation cohorts risks hardcoding systemic measurement artifacts into downstream clinical biomarker pipelines [20,22].

Taken together, these findings indicate that the operational advantages of high-multiplex platforms, particularly throughput, sample efficiency, and breadth, do not necessarily translate into equivalent molecular specificity or cross-platform reproducibility. High-multiplex platforms can efficiently prioritize candidate proteins and generate testable associations at population scale, but their added value is therefore greatest when the study objective prioritizes scalable relative profiling or targeted sensitivity, whereas applications requiring definitive proteoform assignment or cross-platform quantitative equivalence require additional orthogonal validation.

## 4. Strategic Selection Framework

Navigating platform deployment requires aligning the primary research objective with distinct technical boundaries rather than prioritizing nominal protein target counts alone. Relevant analytical dimensions include whether the study design is exploratory or confirmatory, the required sensitivity thresholds and proteome breadth, available sample volume, clinical cohort sizes, the necessity for peptide- or proteoform-level resolution, cross-platform harmonization limits, bioinformatic resource availability, and overall budgetary allocations [47]. Figure 5 illustrates a concentric strategic selection pipeline designed to map these structural priorities directly onto the optimal operational capabilities and inherent constraints characterizing each platform.

Importantly, broad candidate identification should not be equated with definitive biomarker qualification or therapeutic target validation; the latter requires greater analytical and biological resolution, including evidence of specificity, reproducibility, causal relevance, and, where appropriate, proteoform-level characterization.

For large-scale biomarker discovery in clinical cohorts or biobanks where broad affinity-based coverage and standardized throughput are priorities, ultra-high-multiplex aptamer arrays such as SomaScan represent a strong option because current configurations provide approximately 11,000 protein measurements per sample [27]. The principal trade-off is that breadth remains limited to validated aptamer targets and that individual measurements may be influenced by affinity-related effects [39].

When low sample volume and dual-antibody recognition are major priorities, proximity-based assays such as Olink provide well-balanced affinity-based alternative. Its PEA architecture offers high multiplexing with a dual-recognition specificity filter, making it particularly suitable for population studies focused on circulating signaling, inflammatory, cardiovascular, and other low-abundance proteins [16].

For studies prioritizing reagent-independent discovery, peptide-level evidence, or investigation of protein-altering variants, nanoparticle-assisted MS platforms can provide complementary information that is inaccessible to fixed affinity menus [14,29]. This advantage should be weighed against nanoparticle-enrichment bias, MS infrastructure requirements, and the fact that conventional bottom-up workflows do not directly preserve intact proteoform connectivity [18].

For targeted confirmation of a small number of very low-abundance proteins, digital single-molecule immunoassays are often more appropriate than a discovery-scale platform [34]. Their strength is analytical sensitivity rather than proteome breadth, making them well suited to verification studies and clinical contexts in which low circulating concentrations are the principal analytical barrier [17,33,34].

Across clinical domains, the most informative strategy may therefore be staged rather than platform-exclusive. Broad profiling can be used to identify candidate signatures, followed by orthogonal confirmation using an analytically distinct method. This is particularly relevant in cardiovascular, renal, oncological, and neurological biomarker research, where disease-associated proteins may span very different concentration ranges and where biological interpretation can be affected by platform-specific recognition chemistry [38,48,49].

## 5. Current Bottlenecks and Harmonization Challenges

Next-generation high-multiplex proteomics frameworks are undergoing rapid technological evolution, yet universal standardizations for cross-platform calibration, reporting metrics, and validation pipelines remain incomplete [50]. These regulatory and analytical deficits limit straightforward study replication and broad meta-analyses because each platform measures a partly overlapping yet biochemically distinct analytical projection of the circulating biofluid proteome [51]. Consequently, driving progress towards robust clinical translation requires shifting focus away from raw analytical target depth alone, instead prioritizing technical reproducibility, multi-matrix orthogonal confirmation, transparent data reporting, and the systemic interoperability of downstream data structures [22,35].

### 5.1. Reconciling Affinity-Based Epitope Effects and Bioinformatic Silos

Measurements of the identical nominal protein across high-multiplex platforms can vary substantially. In the Atherosclerosis Risk in Communities (ARIC) study, for example, direct head-to-head evaluation of 417 overlapping Olink and SomaScan assays demonstrated a median Spearman rank correlation coefficient of approximately 0.46, accompanied by extreme protein-to-protein variation [44]. Larger multi-platform and proteogenomic evaluations consistently establish that cross-platform concordance remains highly target-specific rather than a fixed property of a given technology [52,53]. Consequently, a candidate biomarker signature discovered via one specific platform cannot be assumed to transfer directly to alternative testing frameworks without rigorous assay-level cross-calibration [20].

These quantitative discrepancies arise because divergent high-throughput configurations profile fundamentally distinct biochemical signals. While the Olink PEA and Quanterix Simoa rely heavily on conformational antibody sandwich recognition, the SomaScan architecture utilizes chemically modified single-stranded DNA aptamers, and the nanoparticle corona adsorption framework couples physicochemical kinetics to bottom-up MS [27,29,48]. Complex biofluid matrix compositions, variable pre-analytical handling protocols, epitope accessibility masking, affinity dissociation kinetics, circulating protein isoforms, and peptide-level ionization characteristics can therefore severely alter the observed analytical signal, even when the underlying native biological abundance remains identical [18,20].

Genetically driven structural alterations provide a critical mechanistic window into these measurement defects. A *cis*-pQTL can reflect a true physiological change in circulating protein concentration, a platform-specific alteration in reagent binding affinity (epitope effect), or a combination of both [46,47]. Large-scale proteogenomic comparative maps show that a substantial fraction of these genetic signals fail to replicate across alternative platforms due to localized binding artifacts [54]. Recent evidence from nanoparticle-enriched MS workflows further highlights the critical utility of reagent-independent, peptide-level mass mapping to systematically differentiate total abundance shifts from localized epitope binding artifacts [14,31]. This resolution is particularly urgent when utilizing high-multiplex screening arrays to prioritize therapeutic targets.

### 5.2. Overcoming Affinity Structural Blindness and Peptide-Level Data Aggregation

While the human genome contains approximately 20,000 protein-coding genes, downstream alternative splicing, sequence variation, proteolytic processing, and post-translational modifications (PTMs) generate an extensively larger pool of distinct circulating proteoforms [19,55]. Current high-multiplex affinity platforms are primarily engineered to recognize predefined macro-level protein targets; consequently, they fail to resolve specific structural isoforms or post-translational states unless affinity reagents were explicitly developed to target those localized features [55,56]. A prominent example of this analytical limitation is cardiac troponin I (cTnI), a vital biomarker for myocardial infarction. In circulation, cTnI exists as a highly heterogeneous mixture of intact proteins, specific proteolytic cleavage fragments, various phosphorylation states, and oxidized complexes. Because standard multiplex screening tools rely on macro-level capture, they generate a single aggregate quantitative signal that averages out these distinct structural modifications, masking the specific degradation kinetics that track true clinical disease progression [57]. A similar issue compromises neurodegenerative profiling of the tau protein; while high-multiplex screening registers total tau levels, clinical utility in Alzheimer’s disease relies on capturing hyperphosphorylated proteoforms at highly localized residue coordinates (e.g., p-tau181, p-tau217, or p-tau231) [58]. Furthermore, for targets like apolipoprotein E (ApoE), common genetic polymorphisms result in distinct isoforms (ApoE2, ApoE3, ApoE4) differing by single amino acid substitutions that fundamentally dictate Alzheimer’s risk. Standard high-multiplex configurations lack the localized structural resolution to differentiate these highly consequential variant domains from baseline protein concentration shifts, illustrating why independent, top-down molecular confirmation remains essential [59,60,61].

### 5.3. Critical Appraisal of Top-Down and Middle-Down Proteomics Constraints

Characterizing intact proteins via top-down proteomics (TDP) and middle-down proteomics (MDP) is frequently framed as a direct remedy for bottom-up inference ambiguity [62]; however, their complementarity should be understood in terms of analytical information rather than as a straightforward solution to the limitations of conventional workflows. TDP strategies are broadly divided into two distinct paradigms—Mass Spectrometry-intensive Top-Down Proteomics (MSi-TDP) and Integrated Top-Down Proteomics (iTDP)—each characterized by unique performance trade-offs regarding molecular weight boundaries, proteome coverage, and throughput [62].

In mainstream MSi-TDP, intact proteoform extracts are resolved directly via liquid chromatography or capillary electrophoresis before gas-phase mass spectrometry analysis. This approach encompasses two main operational variants: denatured top-down proteomics (d-TDP) and native top-down proteomics (n-TDP) [63]. In d-TDP, protein tertiary structures are chemically unfolded to maximize backbone accessibility for gas-phase fragmentation, though this sacrifices native conformation and noncovalent connectivity. Conversely, n-TDP introduces intact protein assemblies into the instrument using soft, non-denaturing volatile salt matrices to preserve noncovalent interactions and ligand configurations. However, both MSi-TDP formats are heavily constrained by instrumental factors. They suffer from severe spectral congestion due to highly charged envelope ions and experience a sharp decline in ion transmission efficiency as macromolecular weight increases. Consequently, broad proteome-scale MSi-TDP workflows remain largely restricted to lower-molecular-weight proteoforms below approximately 30–50 kDa [64].

Integrated Top-Down Proteomics (iTDP) effectively circumvents these molecular weight limitations by incorporating top-front, high-resolution quantitative two-dimensional gel electrophoresis (2DE) tightly coupled with downstream LC-MS/MS characterization. Because iTDP relies on gel-based physical isolation prior to mass spectrometry, it handles complex high-molecular-weight species exceeding 100 kDa without suffering from the spectral congestion or raw gas-phase ion transmission failures that limit MSi-TDP [65]. This enables iTDP to deliver exceptional proteoform-level resolution and sequence coverage across a wide molecular scale.

The primary constraint of iTDP is its highly restricted sample throughput, which prevents its direct application as a primary screening tool in population-scale clinical cohorts. Nonetheless, its ability to achieve total intact proteoform resolution means it serves as an ideal analytical complement to high-throughput discovery platforms. Rather than deploying these tools in isolation, low-throughput iTDP can be utilized as a targeted validation checkpoint. By resolving structural variants, localized post-translational modifications, and intact protein connectivity, iTDP can complement and orthogonally verify the aggregate candidate biomarker signatures prioritized by high-multiplex affinity and nanoparticle corona screening workflows.

### 5.4. Consolidated Strategic Guidelines for Research Practices

To successfully translate these multi-tiered analytical observations into reproducible clinical practices, study designs should deploy an integrated framework that systematically bridges these individual technological limitations:

1. Reference Control Calibration: Establishing a standardized, multi-matrix reference biofluid control across the international proteomics community is required to resolve data discrepancies. Interleaving these benchmark controls within high-multiplex cohort studies permits the generation of platform-specific calibration curves, enabling mathematical normalization and robust cross-study comparisons [66,67].
2. Epitope-Aware pQTL Pipelines: Incorporating “epitope-aware” filtering into genetic mapping workflows is essential to flag structural variants that distort affinity-reagent kinetics. Cross-referencing identified pQTLs against localized protein structure isolates true abundance changes from false-positive binding artifacts [68].
3. Synergistic Multi-Tiered Workflows: Rather than viewing affinity platforms and discovery mass spectrometry as mutually exclusive systems, translational study designs must integrate them into multi-tiered analytical pipelines. High-throughput discovery should deploy ultra-high-multiplex affinity platforms (Olink or SomaScan) for broad front-end screening across massive clinical biobanks to nominate candidate disease networks. This screening phase is then paired with nanoparticle-driven discovery mass spectrometry on a selected, lower-throughput cohort subset to systematically map peptide boundaries and localized PTM layouts of prioritized biomarker targets [69].
4. Bounded Top-Down Validation Pipelines: Resolving the bottom-up protein-inference problem requires development in instrument architectures that preserve long-range molecular connectivity. While intact-mass and middle-down mass spectrometry fragmentation variants remain constrained by lower throughput in plasma applications and by challenges in the transmission and analysis of higher-molecular-weight species, their future integration with front-end nanoparticle enrichment arrays holds targeted validation utility. Utilizing these intact-mass workflows strictly as low-throughput validation checkpoints on selected targets allows investigators to bypass tryptic digestion artifact boundaries, isolating structural, disease-specific proteoforms prior to downstream clinical deployment [61].

Ultimately, identifying highly selective biomarkers and valid therapeutic drug targets requires a shift away from single-platform evaluation toward an integrated, multi-stage translational roadmap. As illustrated in the strategic pipeline, this optimal workflow begins with high-throughput screening using ultra-high-multiplex affinity arrays to cast a wide net across large clinical cohorts, efficiently prioritizing candidate protein networks associated with a disease phenotype. However, because these front-end readouts yield aggregate signals prone to epitope masking and proteoform averaging, they cannot serve as definitive molecular endpoints. The next critical stage demands narrowing the cohort focus to integrate nanoparticle-assisted bottom-up mass spectrometry, which provides open-discovery, peptide-level sequence mapping to rule out genetic binding artifacts (such as pQTL variations) [14]. Finally, the workflow must be completed by deploying low-throughput, high-resolution intact proteoform characterization methods, such as Integrated Top-Down Proteomics (iTDP) or Middle-Down Proteomics (MDP), on highly curated sample subsets [19]. By preserving physical macromolecular connectivity, this terminal validation stage isolates the exact disease-specific proteoforms (e.g., specific cleavage variants or hyperphosphorylated states) responsible for driving pathological mechanisms [69]. This multi-tiered paradigm ensures that prioritized candidates undergo a rigorous transition from high-throughput relative screening to definitive, structure-informed molecular confirmation before entering costly clinical development pipelines.

## 6. Conclusions

High-multiplex proteomics technologies enable population-scale profiling, but their quantitative readouts are not interchangeable. Because platforms differ fundamentally in recognition chemistry, sensitivity, and throughput, platform selection must be driven by the specific research objective rather than raw target counts alone. Broad screening is highly valuable for candidate prioritization, but it should not be equated with definitive biomarker qualification. Establishing biological specificity and proteoform-level identity requires additional analytical evidence. Consequently, a staged strategy combining high-multiplex relative profiling with targeted orthogonal methods remains the most robust choice when structural resolution becomes critical. Translating high-multiplex discovery into clinical utility requires structured data harmonization and multi-tiered validation. Implementing multi-matrix reference standards, epitope-aware proteogenomic filters, and intact-mass workflows provides a rigorous framework to resolve structural isoforms and post-translational modifications. Ultimately, clinical translation requires moving beyond raw measurement depth alone, ensuring that captured analytical signals are reproducible, structurally interpretable, and transferable across distinct platforms.

## Figures and Tables

**Figure 1:**
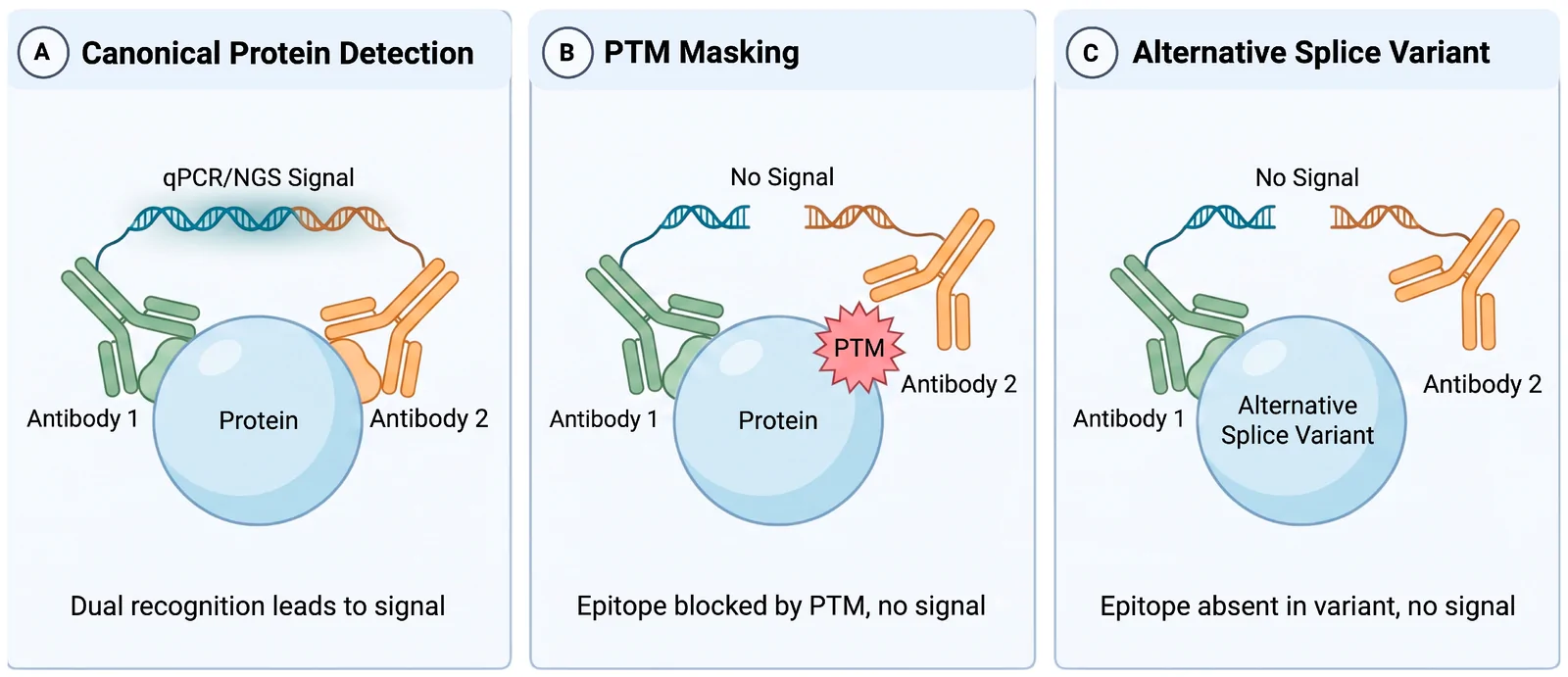
Architectural Mechanisms and Structural Constraints of the Proximity Extension Assay (PEA). Productive signal generation requires simultaneous dual-epitope binding by Antibody 1 and Antibody 2 to permit oligonucleotide hybridization and extension (**A**). While minimizing cross-reactivity, this configuration introduces a systemic vulnerability to structural protein variation. Post-translational modifications (PTMs) can physically block antibody binding, resulting in a loss of reporter signal despite unchanged total protein abundance (**B**). Similarly, alternative splicing events that omit an engineered target exon preclude dual-antibody assembly and suppress signal generation (**C**). PEA readouts are therefore governed by localized epitope accessibility rather than providing an absolute quantification of all circulating proteoforms.

**Figure 2:**
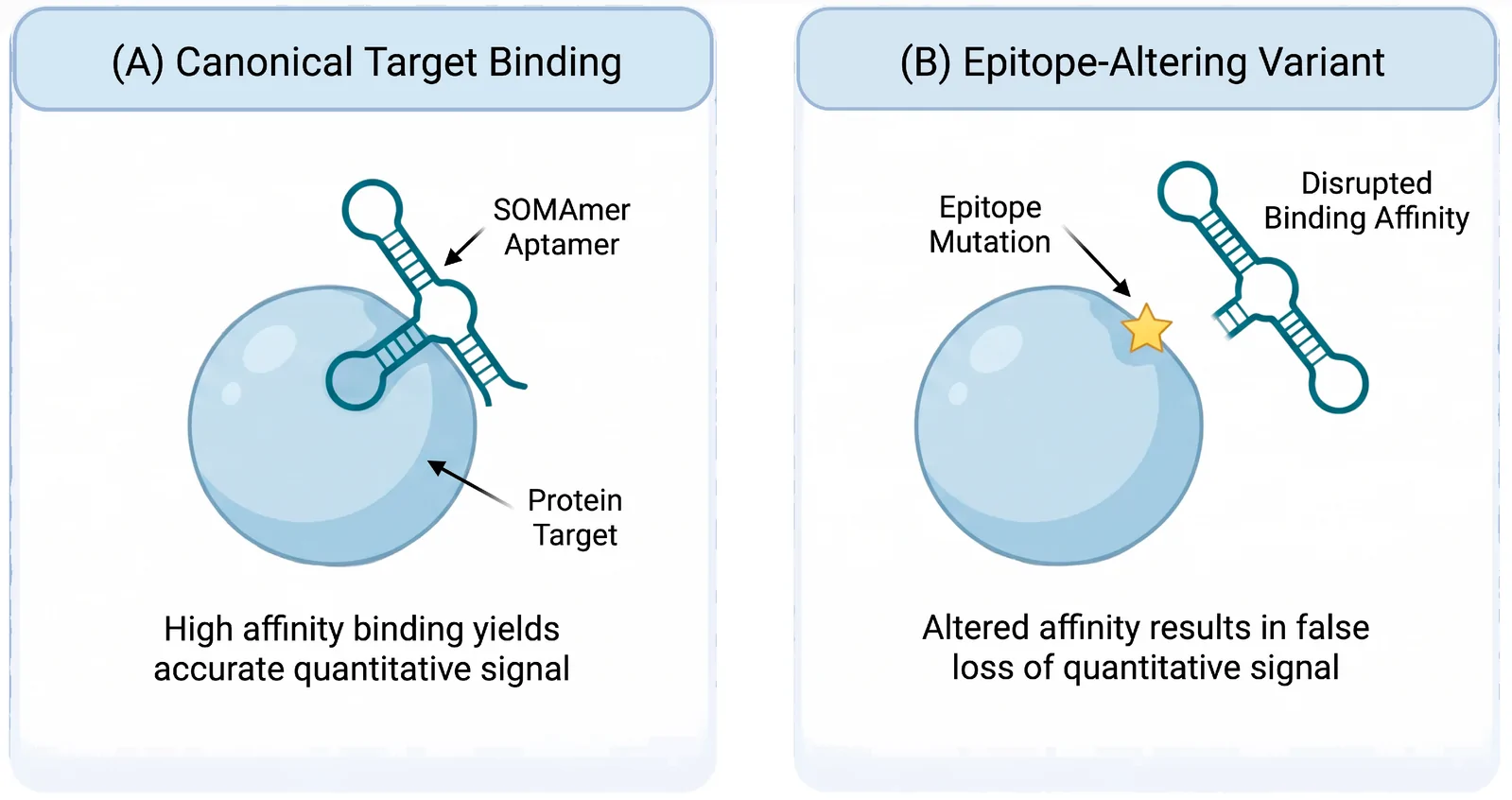
Biochemical Architecture and Genetic Susceptibility of the Aptamer-Based SomaScan Assay. Synthetic, chemically modified single-stranded DNA aptamers (SOMAmers) rely on precise three-dimensional structural conformations to bind designated epitopes on target proteins (**A**). While this parallelized framework provides exceptional multiplexing capacity, its quantitative accuracy remains highly dependent on genetic sequence conservation. A protein-altering variant (such as a single amino acid substitution or structural mutation) directly within the targeting region can structurally distort the binding epitope, altering dissociation kinetics and reducing SOMAmer affinity (**B**). Consequently, the subsequent denaturing elution and microarray/NGS readout register a false decrease in signal intensity, despite unchanged circulating levels of the protein concentration.

**Figure 3:**
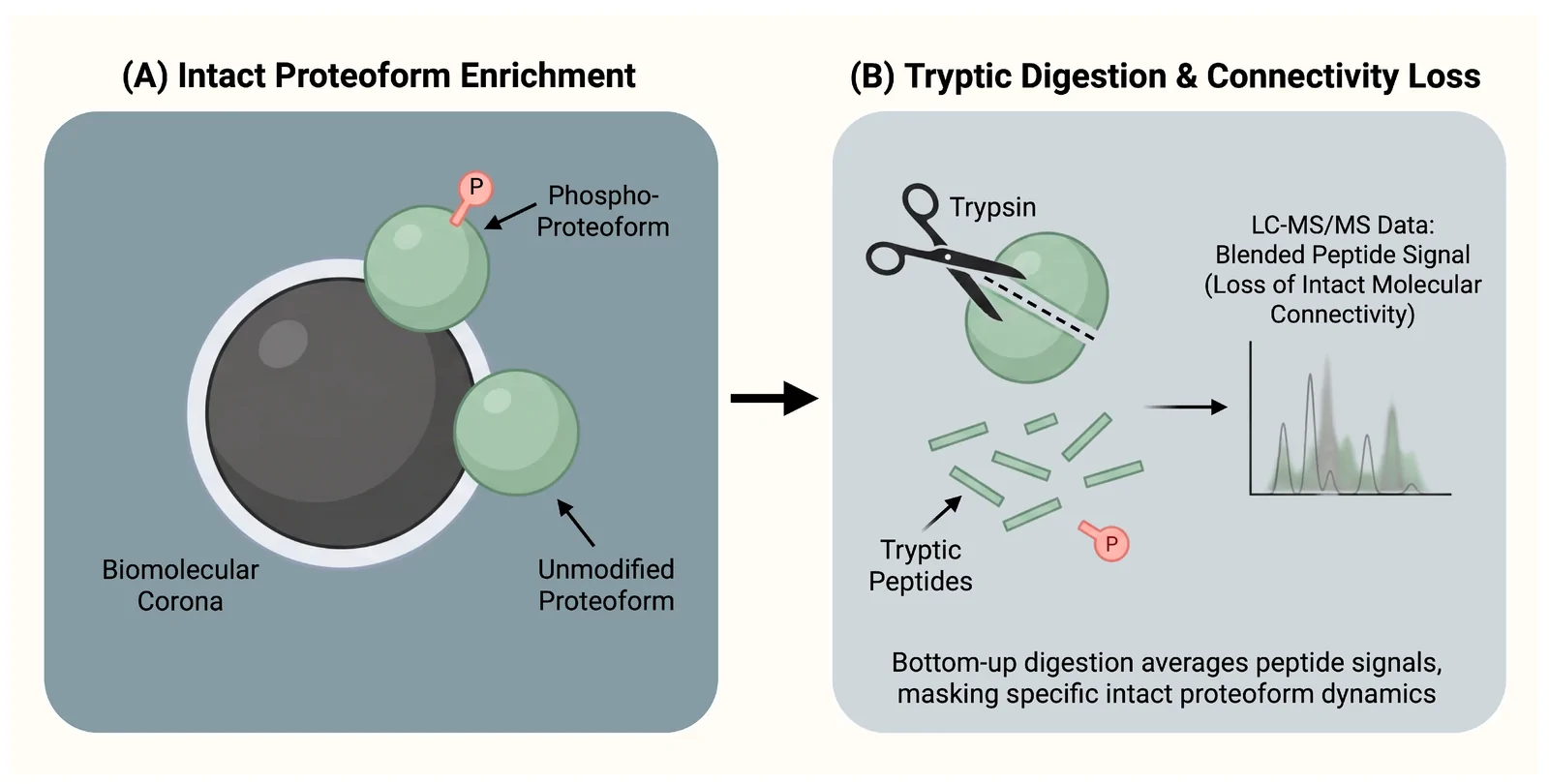
Nanoparticle Corona Enrichment and Intact Connectivity Loss on the Seer Proteograph Platform. Physicochemical adsorption drives the formation of a biomolecular corona on engineered nanoparticle surfaces, capturing distinct intact protein variants (**A**). However, downstream bottom-up mass spectrometry workflows require enzymatic cleavage. Tryptic digestion breaks the intact proteins into short peptide fragments, physically separating post-translational modifications (PTMs) or structural domains from their original macromolecular sequence (**B**). The resulting LC-MS/MS readout measures an average, blended peptide signal, which compromises the direct reconstruction of the parent proteoform. Consequently, while reagent-independent, this framework faces systemic constraints in distinguishing specific proteoform dynamics from overall changes in total protein abundance.

**Figure 4:**
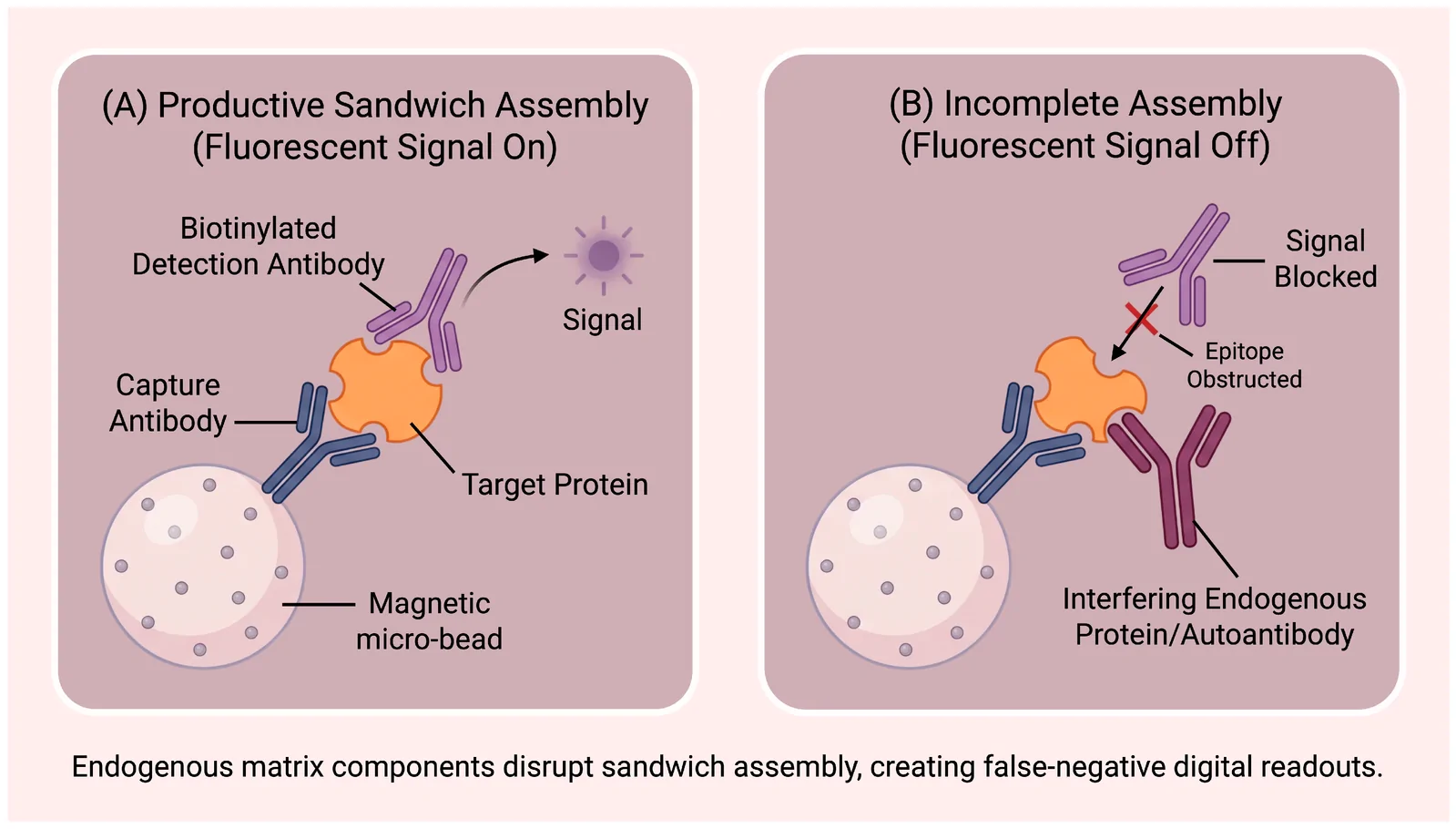
Digital Immunoassay Architecture and Matrix Vulnerabilities in Single-Molecule Arrays. Miniaturized femtoliter-scale arrays enable the digital counting of single-molecule target proteins captured via specific sandwich immunoassay pairs (**A**). While providing sub-picogram analytical sensitivity, this platform remains fundamentally reliant on dual-antibody epitope accessibility. Endogenous biofluid components, such as circulating autoantibodies, heterophilic antibodies, or matrix interference factors, can sterically hinder or competitively block the secondary detection antibody from binding its designated epitope (**B**). This disruption prevents the formation of the enzyme-labeled complex, causing the micro-well to register an artificial “Off” fluorescent state. Consequently, in complex clinical biofluids, digital readouts are prone to structural masking effects that obscure the true abundance of the target protein.

**Figure 5:**
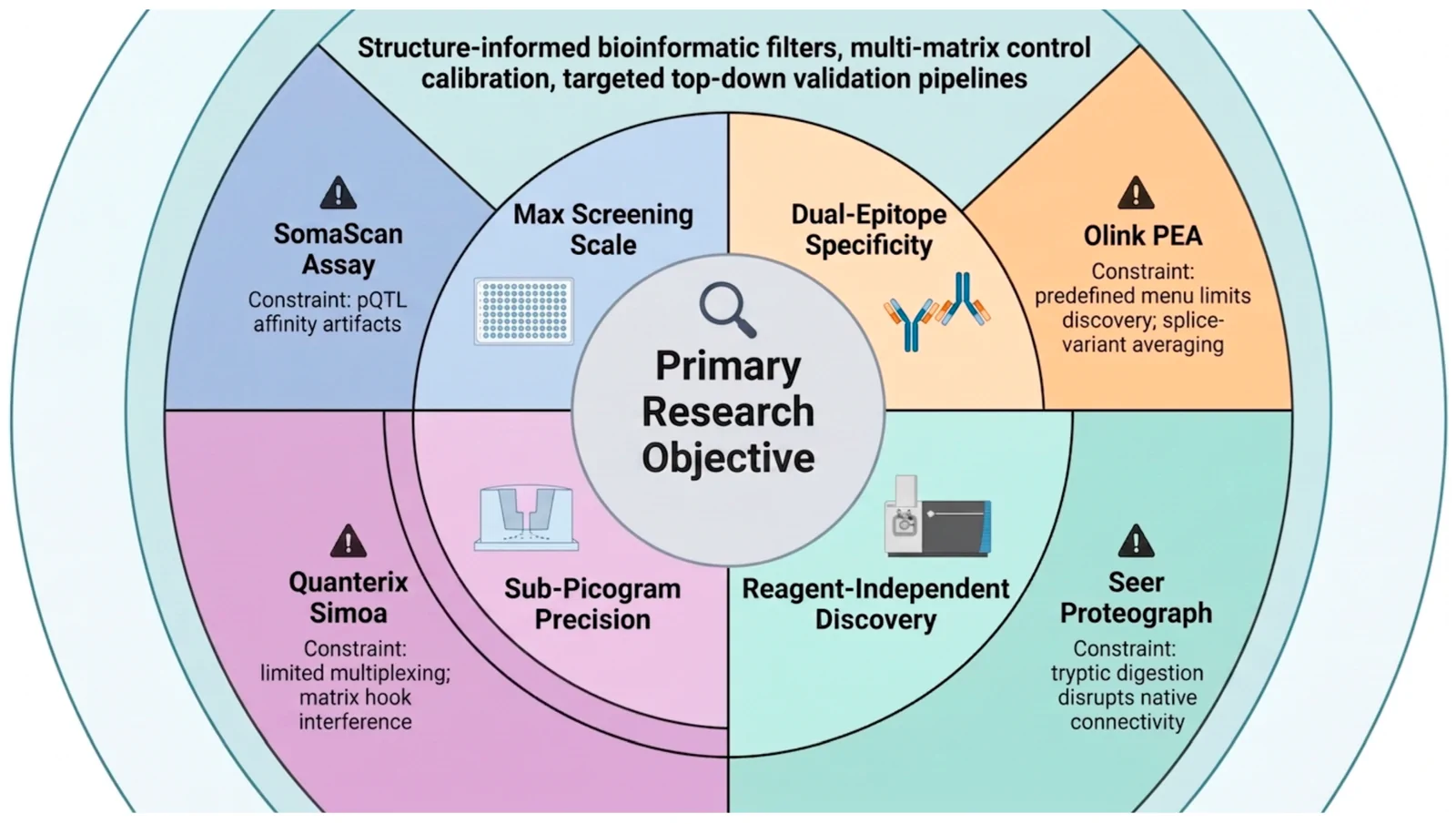
Strategic Selection Pipeline for Biofluid Proteomics Platforms. Navigating clinical proteomics requires aligning the primary research objective (central core) with a tailored operational workflow (radiating wedges). Rather than serving as interchangeable tools, each platform may serve a distinct role in an integrated analytical pipeline: SomaScan and Olink are suited to high-throughput biobank screening and relative pathway characterization; the Seer Proteograph enables reagent-independent, variant-aware peptide discovery; and Quanterix Simoa provides ultra-sensitive, sub-picogram targeted clinical verification. To achieve robust translation, all candidate biomarkers should undergo structure-informed filtering, multi-matrix reference calibration, and orthogonal validation workflows before definitive biological or clinical interpretation.

**Table 1:** Comparative Structural and Operational Parameters of High-Multiplex Proteomics Platforms [10,11,12,13].

Characteristic	Olink PEA	SomaScan	Nanoparticle Corona LC-MS	Digital Single-Molecule Immunoassays
Recognition principle	Dual-antibody proximity extension assay	Chemically modified DNA aptamers (SOMAmers)	Nanoparticle corona enrichment followed by LC-MS/MS (e.g., Seer Proteograph Suite)	Digital single-molecule sandwich immunoassay
Representative multiplexing	5400 + proteins (Explore HT)	~11,000 protein targets (11K Assay)	Mass spectrometry-dependent; typically, thousands of protein groups	Low-plex and assay-dependent; typical bead panels are low-plex; selected planar arrays up to 10-plex
Representative sample input	~2 µL plasma/serum (Explore HT)	~55 µL (11K Assay)	~240 µL neat plasma in standard workflow; micro-volume protocols available	Assay-dependent; commonly tens of microliters
Primary readout	Relative protein-target signal from amplified DNA reporters	Relative protein-target signal from SOMAmer surrogates	Peptide-level tandem mass spectrometry (LC-MS/MS) signal	Digital single-molecule protein fluorescent counting signal
Primary operational advantage	Low sample-volume consumption paired with high multiplexing scale	Broadest available affinity-based menu density and screening scalability	Reagent-independent discovery, variant mapping, and direct peptide evidence	Exceptional sub-picogram analytical sensitivity for targeted low-plex markers
Main architectural constraint	Menu-bound; aggregate signals are vulnerable to epitope and matrix interference	Menu-bound; aggregate signals are susceptible to structural variant binding artifacts	Physicochemical corona adsorption bias and structural connectivity loss via bottom-up digestion	Highly restricted multiplexing breath and vulnerability to matrix hook effects
Optimal pipeline deployment	Volume-constrained large cohorts and relative pathway profiling	Population-scale relative biomarker screening and network discovery	Untargeted structural variant discovery, isoform mapping, and reagent-free profiling	Precise targeted clinical verification of ultra-low-abundance biomarkers
Typical workflow role	Discovery screening/Multi-omic association	Discovery screening/Multi-omic association	Discovery exploration/Structural characterization	Target verification/Clinical biomarker validation

## Data Availability

No new data were created or analyzed in this study. Data sharing is not applicable to this article.
